# Ultrasound of Small Bowel Obstruction: A Pictorial Review

**DOI:** 10.3390/diagnostics11040617

**Published:** 2021-03-30

**Authors:** Nicola Rosano, Luigi Gallo, Giuseppe Mercogliano, Pasquale Quassone, Ornella Picascia, Marco Catalano, Antonella Pesce, Valeria Fiorini, Ida Pelella, Giuliana Vespere, Marina Romano, Pasquale Tammaro, Ester Marra, Gabriella Oliva, Marina Lugarà, Mario Scuderi, Stefania Tamburrini, Ines Marano

**Affiliations:** 1 Department of Radiology, Ospedale del Mare, ASL NA1 Centro, 80147 Napoli, Italy; marco26catalano@yahoo.it (M.C.); antonellapesce1986@libero.it (A.P.); valeria.fiorini@libero.it (V.F.); ida.pelella@virgilio.it (I.P.); ines.marano@tiscali.it (I.M.); 2 Department of Radiology, Università degli Studi della Campania Luigi Vanvitelli, 80138 Napoli, Italy; luigigallo992@gmail.com (L.G.); pasquale.quassone@gmail.com (P.Q.); ornellapicascia@gmail.com (O.P.); 3 Department of Radiology, University of Naples “Federico II”, 80131 Napoli, Italy; giuse.mercogliano@gmail.com; 4 Department of Gastroenterology, Ospedale del Mare, ASL NA1 Centro, 80147 Napoli, Italy; giuvespere@gmail.com; 5 Department of Surgery, Ospedale del Mare, ASL NA1 Centro, 80147 Napoli, Italy; marinaromano.na@gmail.com (M.R.); pasqualetammaro81@gmail.com (P.T.); 6 Department of Surgery, University of Naples “Federico II”, 80131 Napoli, Italy; estermarra9@gmail.com; 7 Department of Internal Medicine, Ospedale del Mare, ASL NA1 Centro, 80147 Napoli, Italy; gabrioliva@icloud.com (G.O.); marinalugara82@gmail.com (M.L.); 8 Department of Emergency A.O.E. Cannizzaro, 95126 Catania, Italy; mscuderi@tiscali.it

**Keywords:** bowel ultrasound, small bowel obstruction, emergency ultrasound

## Abstract

Small bowel obstruction (SBO) is a common condition requiring urgent attention that may involve surgical treatment. Imaging is essential for the diagnosis and characterization of SBO because the clinical presentation and results of laboratory tests may be nonspecific. Ultrasound is an excellent initial imaging modality for assisting physicians in the rapid and accurate diagnosis of a variety of pathologies to expedite management. In the case of SBO diagnosis, ultrasound has an overall sensitivity of 92% (95% CI: 89–95%) and specificity of 93% (95% CI: 85–97%); the aim of this review is to examine the criteria for the diagnosis of SBO by ultrasound, which can be divided into diagnostic and staging criteria. The diagnostic criteria include the presence of dilated loops and abnormal peristalsis, while the staging criteria are represented by parietal and valvulae conniventes alterations and by the presence of free extraluminal fluid. Ultrasound has reasonably high accuracy compared to computed tomography (CT) scanning and may substantially decrease the time to diagnosis; moreover, ultrasound is also widely used in the monitoring and follow-up of patients undergoing conservative treatment, allowing the assessment of loop distension and the resumption of peristalsis.

## 1. Introduction

Small bowel obstruction is a common disease; its incidence in patients who present to the emergency department (ED) is estimated at 2–8%, and about 15% of these patients are admitted to the surgical unit [1,2,3]. The term ‘ileus’ refers to partial or complete blockage of the progression of solid, liquid, and gaseous material within the intestinal lumen. Based on the level of obstruction, a bowel obstruction can be classified as small bowel obstruction (SBO) or large bowel obstruction (LBO); SBO is usually caused by benign lesions, whereas LBO is often caused by cancer. In addition, SBO may be proximal (high SBO) or distal (low SBO) [4]. An ileus may be paralytic/adynamic or mechanical. In paralytic ileus, there is no physical cause for the obstruction of intestinal transit, whereas, in mechanical ileus, there is a loss of normal lumen patency which can be complete or incomplete. The cause of mechanical obstruction can be intraluminal (gallstones, vegetative tumors, or foreign bodies), intramural (stenosis generated by parietal infiltration of the tumoral process or by an inflammatory reaction, e.g., Crohn’s disease), or extraintestinal (bands, adhesions, carcinosis, retro/intraperitoneal mass). The most frequent cause of small bowel occlusion is adhesions, followed by hernias and neoplasm; in developing countries, infectious causes (such as abdominal tuberculosis) should be considered [5,6,7,8,9]. Although adhesions resulting from prior abdominal surgery are the predominant cause of SBO, accounting for 60% to 75% of cases, SBO should not be excluded in patients without any history of previous surgical procedure (‘virgin abdomen’). In this subgroup of patients, SBO has been reported at a frequency of 7.7–13.7% and is caused by de novo adhesions (54%) and neoplasms [8,10]. The hallmarks of intestinal obstruction include colicky abdominal pain, nausea, vomiting, abdominal distension, and cessation of flatus and bowel movements. The presence and severity of symptoms vary based on the acuity of the obstruction and its anatomic location, ranging from vague symptoms such as epigastralgia and nausea to more severe and typical patterns with constipation intense vomiting, consequent dehydration, and hypochloremic metabolic alkalosis. In the case of high obstructions, there is an early onset of gastric/biliary vomiting, whereas vomiting appears late and can be fecal in the case of low obstructions. Considering that SBO often occurs as an array of symptoms, a diagnosis based only on clinical presentation may not be well-grounded. Small bowel mechanical ileus is considered a developmental and time-dependent disease, and early diagnosis is fundamental in avoiding complications such as vascular compromise or perforation. Small bowel occlusion is classified into simple, decompensated, or complicated ileus depending on the absence/presence of loop decompensation signs [11,12]. Simple mechanical ileus represents the first stage of occlusion, and it is characterized by the presence of a distended bowel upstream of the obstructive fulcrum, with gaseous or, more often, fluid/gaseous stasis; in this phase, the upstream loop shows hyper-kinesis and hyper-representation of valvulae conniventes. This appearance reflects the bowel’s propulsive attempt to overcome the obstacle; the downstream loops instead present normal or reduced caliber. Since there is no alteration in vascular circulation during the early/simple stages, the patient is subjected to conservative therapy and monitoring, aimed at determining the grade of loop distension with consequent reduction, firstly of intraluminal stagnation, and secondly by caliber with possible complete resolution. Resolution of a simple ileus in stable patients is attempted with non-operative management (NOM) from 48 to 72 h with hydroelectrolytic support; the oral intake is restricted and nasogastric intubation is usually performed for decompression in most patients [13,14]. If conservative therapy fails or hydroelectrolytic alterations appear, surgical treatment is carried out. With the persistence of the obstruction, the upstream loop of the obstructive fulcrum becomes exhausted and loses its enteric fluid resorption capacity with consequent alteration of the parietal microcirculation and oozing of fluid, firstly in the recesses between the loops and then in the mesenteric fat. At this stage, the SBO is defined as decompensated. In a decompensated ileus, free fluid between bowel loops represents the first sign of loop decompensation. Intestinal walls appear thin and kinesis is reduced or ineffective, characterized by a back-and-forth movement. In those patients, the temporal window of the NOM is narrowed, and the patient should be evaluated for surgery. In select patients with adhesive or partial SBO, oral administration of hypertonic water-soluble contrast media may have therapeutic effects and assist in resolution [13,14,15]. Complicated ileus represents a surgical emergency and is characterized by loop vascular distress with consequent vascular damage, necrosis, and subsequent bowel perforation with a high possibility of peritonitis and potentially fatal evolution. In this stage, the upstream loops of the obstructive fulcrum are akinetic with parietal and valvulae conniventes thickening due to vascular infarction. Decompensated ileus and closed-loop obstruction, in which a segment of the bowel is obstructed proximally and distally, are considered surgical emergencies [16,17,18]. The complication risks of intestinal occlusion are very high: strangulation occurs in 30% of cases and bowel necrosis in 15%, and both may lead to perforation, sepsis, and death. Risk factors for complicated SBO include age, comorbidities, and a delayed diagnosis. The high rate of complications and the need for early diagnosis and treatment necessitate prompt diagnosis and staging [2,19]. In the case of suspected bowel obstruction, imaging is essential because neither the presence nor the absence of clinical features or isolated laboratory findings can exclude or confirm bowel strangulation or necrosis. Fever, leukocytosis, or acidosis represent late systemic signs suggesting that loop necrosis has already occurred. For these reasons, imaging is essential because the clinical presentation and results of laboratory tests may be nonspecific.

Imaging should achieve the following specific aims:

• Confirm or exclude intestinal obstruction (or lead to other differential diagnoses via detection);
• Define the level of obstruction (stomach, jejunum, ileus, or colon);
• Define the cause of the obstruction;
• Define the severity of the obstruction (staging).

The imaging methods used in the evaluation of a patient with suspected bowel obstruction are conventional radiology, ultrasound, computed tomography (CT), and magnetic resonance imaging (MRI). CT is the gold-standard imaging modality in the diagnosis and staging of small bowel obstruction [20,21,22,23,24]. Plain abdominal X-ray in the supine and upright position has a diagnostic accuracy ranging 50–70% and has low specificity; moreover, X-rays can appear normal in patients who have complete, closed-loop, or strangulated obstructions [25,26]. MRI is a reliable method but has some limitations that prevent its wider use in this field, such as long emergency management time, lower availability, higher costs, reduction in image quality with respiratory and bowel movements, and MRI contraindications related to the patient [27,28]. Ultrasound represents a rapid diagnostic modality. Several studies have demonstrated the high diagnostic accuracy of ultrasound in confirming or excluding bowel obstruction, allowing rapid therapeutic classification of the patient and representing a valuable tool in monitoring a patient undergoing conservative treatment. Ultrasound can accurately diagnose SBO, determining the presence or absence of pathology, with no significant difference from CT and limiting the need for CT scanning in the emergency department, speeding up the patient’s management with expeditious admittance to hospital [11,12,29,30,31,32,33]. The majority of stable patients without sonographic signs of parietal damage and with the presence of bowel kinesis are nowadays conservatively managed and can be monitored with a series of ultrasound examinations to evaluate the progress of intestinal obstruction. This paper will review the diagnostic and staging signs of ultrasound in the diagnosis of small bowel obstruction.

## 2. Ultrasound Technique

**Figure 1:**
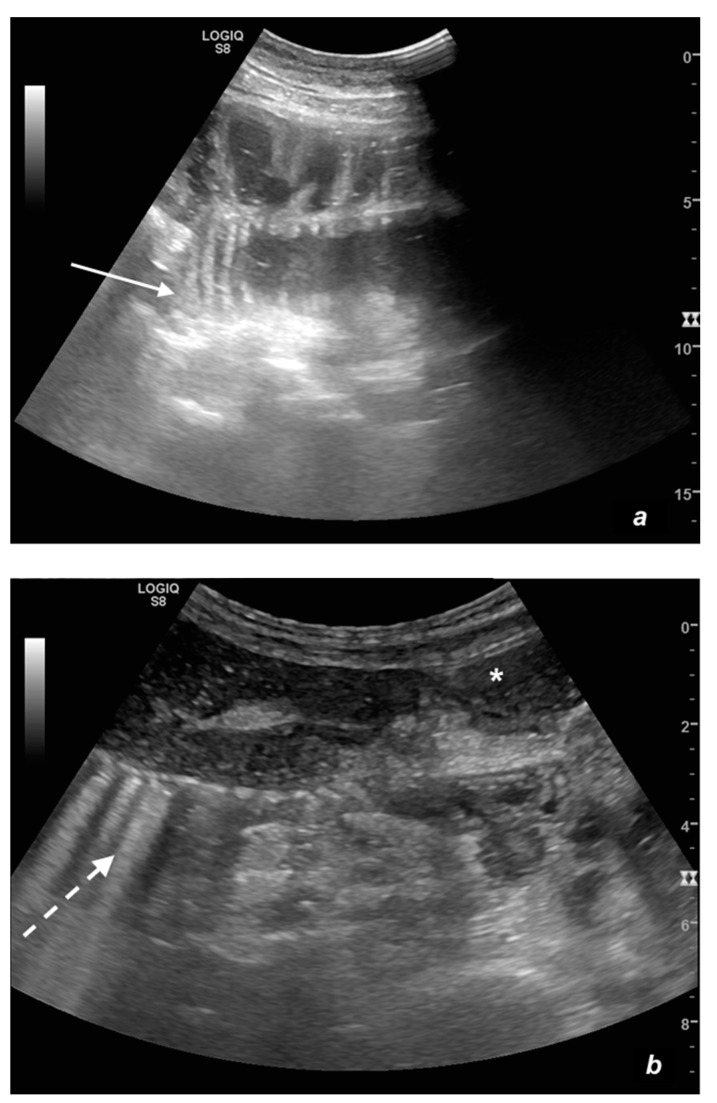
(**a**,**b**). A simple SBO. Ultrasound images show fluid-filled, dilated small bowel loops (**a**,**b**) with hyper-kinesis and hyper-representation of valvulae conniventes (white arrow) (**a**); tail comet artifacts are visible due to air-fluid levels (dashed arrow) (**b**) and groups of bowel loops with severe differences in diameter (‘bowel jump diameter’) are evident more superficially (*) (**b**). No free fluid was detected in the abdominal cavity or between bowel loops.

**Figure 2:**
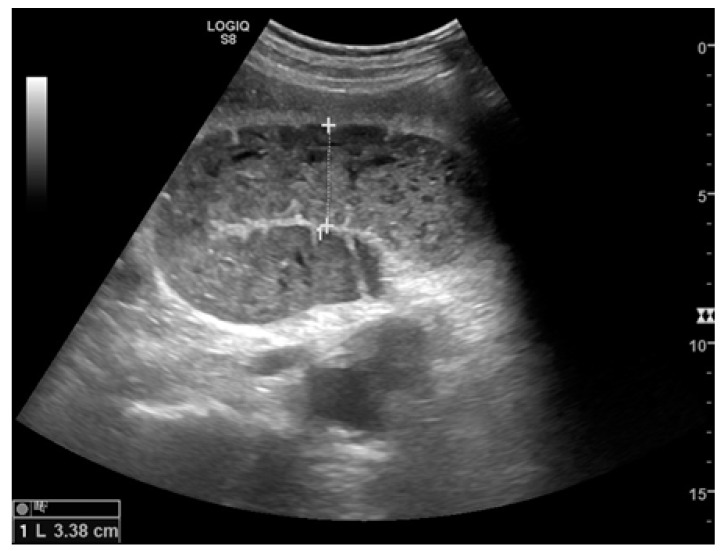
A dilated small bowel loop with a caliber of more than 3 cm (dotted line) with trapped feces defines a ‘small bowel feces sign’. Bowel walls appear thin, and the folds flatten.

The ultrasound examination is performed with the patient in a supine position and spontaneously breathing, using a convex probe (2–6 MHz). A high-frequency linear probe (7–12 MHz) is used to better characterize the state of the loop and show the level of obstruction [34,35]. A complete ultrasound abdominal examination should be performed in order to confirm or exclude the presence of other pathologies (differential diagnosis); this is mandatory in order to avoid ‘satisfaction errors’, which, in the case of an ileus, are not scarce, considering that a mechanical ileus can have extraintestinal causes and paralytic ileus can represent a reaction to other underlying pathologies. The assessment of the intestinal loops is performed via the *Global View* technique [11,12]: a ‘global’ and panoramic assessment of the intestine is performed from the bottom upwards and with vertical probe movements exploring the entire abdomen starting from the right iliac fossa followed by horizontal movements from the right to the left side. The ‘global view’ approach allows for the identification of dilated small bowel loops; in the case of occlusion, it should not be underestimated that bowel loops can be dislocated and not grouped physiologically in the mesogastrium. Once the group of dilated bowel loops is identified, the diameter, kinesis, and parietal and valvulae conniventes thickness are evaluated (Table 1, Figure 1a,b and Figure 2).

**Figure 3:**
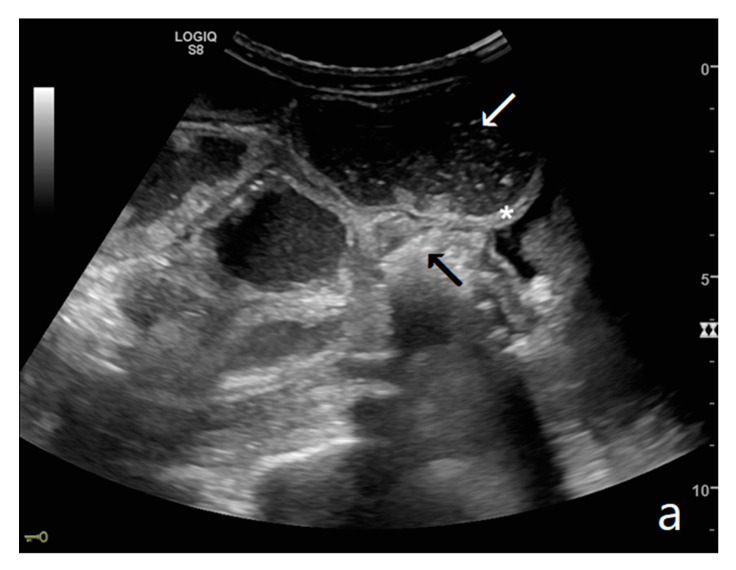

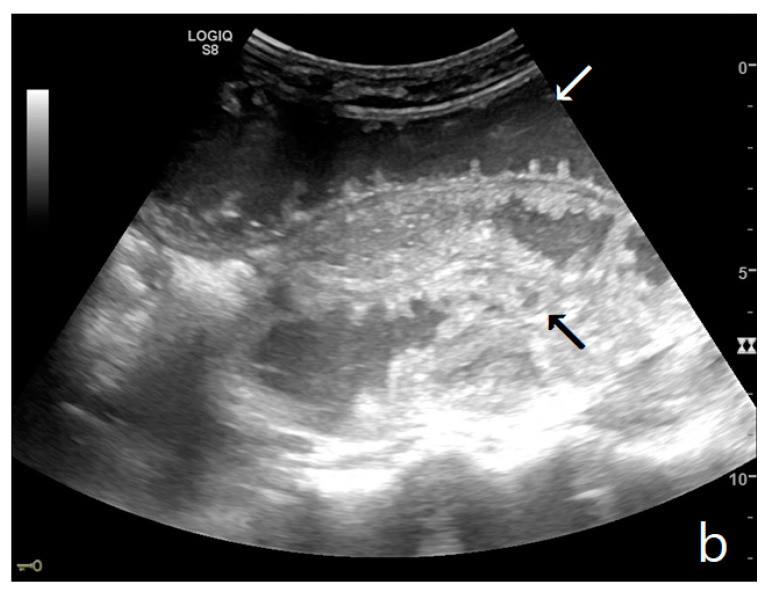
A decompensated SBO, presenting fluid-filled, dilated small bowel loops with increased parietal thickening (*) (**a**) and free fluid between bowel loops (**a**). ‘Caliber jump’: a difference in caliber between the swollen loops upstream (white arrows) (**a**,**b**) and the collapsed loops downstream of the obstruction (black arrows) (**a**,**b**).

The presence or absence of free liquid between the loops should be assessed. At this level, ancillary signs like evidence of groups of bowel loops with severe differences in diameter or kinesis, named bowel jumps, can be identified (Figure 3a,b) [12].

Once the obstructive fulcrum has been identified, it is advisable to try to find the cause of the obstruction, easier in the case of a hernia or extraluminal mass [7], but more difficult and not always possible through ultrasound in the case of adhesions or bridles. The identification of the level of the obstruction point location is possible because the different portions of the intestine can be recognized thanks to their topography and specific morphological criteria. The stomach is identified thanks to its localization and parietal aspects characterized by the more pronounced muscular layer, particularly at the antrum; moreover, in conditions of fluid distension, the mucous folds are clearly evident. The small intestine is characterized by its diameter (much smaller than that of the colon) and by the presence of valvulae conniventes, which gradually reduce in number and height from the proximal jejunum to the distal ileum and are more clearly visible when the loops are spread out by liquid (keyboard sign) [35,36]. Two landmarks are used to study the colon: the first is the cecum and the second is the sigma. Once one of the two points has been identified, the large intestine can be followed in both directions. Ultrasound visualization of the colon is more limited compared to ultrasound visualization of the small intestine; on the left colon, haustra are not always clearly evident, whereas, in the right colon, only the anterior wall is often recognizable since the posterior wall is masked by gas. Ultrasound recognition of the cecum is very important in the evaluation of mechanical ileus to determine the degree of distension, the involvement of the last loop, and the continence of the ileus–cecal valve [11,12,29,34,35].

### Tips and Tricks

There are some interesting tips and tricks that can help in the ultrasound evaluation of SBO [11,12,35]:

Bypass technique: in the case of intestinal obstruction, with the presence of bowel gas distension especially at an early stage of the disease, it may help to bypass the intestinal air by placing the ultrasound probe more laterally to highlight fluid stasis.

Graded compression technique: it may be useful to use graduated compression with a probe in order to improve the quality of the images; this technique is largely used in ultrasound bowel investigation. 

Point of pain: after completing the ultrasound examination with the convex probe, it is very important to ask the patient to locate the point of greatest pain. Through a clinical ultrasound evaluation, guided by the patient’s symptoms, it can be possible to identify the fulcrum and the cause of obstruction.

## 3. Ultrasound Criteria for the Diagnosis of Small Bowel Occlusion

Ultrasound criteria for the diagnosis of SBO can be divided into diagnostic and staging criteria (Table 2) [23]. 

The diagnostic criteria include the presence of dilated loops and abnormal peristalsis while staging criteria are represented by parietal and valvulae conniventes alterations and by the presence of free extraluminal fluid.

### 3.1. Diagnostic Criteria

#### 3.1.1. Loop Dilatation

Small bowel dilatation is defined as bowel diameter ≥2.5 cm measured from outer wall to outer wall [12,35,37]. At an early stage of the disease, the diameter should not be considered an absolute criterion for diagnosis, and other signs must be used: the bowel loop diameter at this stage could be within the normal range, but bowel loops are fluid-filled, hyperkinetic, and with plicar hyper-representation (Figure 1a and Figure 2a) [12].

#### 3.1.2. Kinesis Alteration

Peristalsis alteration represents a fundamental criterion for the diagnosis of mechanical ileus [23]. In an emergency setting, there is no numerical nor quantitative parameter to evaluate kinesis, and although the evaluation is subjective, the method appears to have excellent diagnostic accuracy [12,31,38]. The kinesis can be reduced; ineffective, with a back-and-forth motion; or completely absent. Two important notes regarding the evaluation of peristalsis must be highlighted: the first, already mentioned above, concerns the ileum in the early stage in which the upstream bowel loop may appear hyperkinetic. A simple ileus at an early stage may not be easy to diagnose and requires adequate training, but evidence of fluid stasis, hyperkinesis, and a mildly dilated bowel loop can increase diagnostic confidence. Another important note concerning the evaluation of bowel kinesis is the possible error in interpreting false bowel movements due to the transmission of diaphragm breathing excursion; in this case, the false bowel movement appears to be synchronous with breathing events. In summary, bowel kinesis can be increased when SBO is in the initial phase but is reduced, ineffective, or absent in decompensated and complicated ileus. In the case of the absence of peristalsis, the bowel loop is defined as akinetic. In ultrasound evaluation, evidence of groups of bowel loops with different kinesis and diameter (loops proximal to the obstruction or downstream) increases diagnostic confidence (bowel jump kinesis) [12].

### 3.2. Staging Criteria

#### 3.2.1. Free Fluid

The persistence of obstruction causes an increase in endoluminal pressure, and the liquid content normally present in the intestinal lumen cannot be reabsorbed. Bowel layers act as a sponge, determining the passage of fluid in the peritoneal cavity (Figure 3a,b). In the initial phase, the liquid is disposed between the recesses of the mesenteric fan, giving rise to the characteristic ‘sign of the thong’ [39]. With the persistence of the obstruction, the amount of free fluid increases, and it can be found in the abdominal cavity. The presence of free fluid is directly correlated to bowel parietal vascular alterations [13,37,40,41].

#### 3.2.2. Parietal Alterations

**Figure 4:**
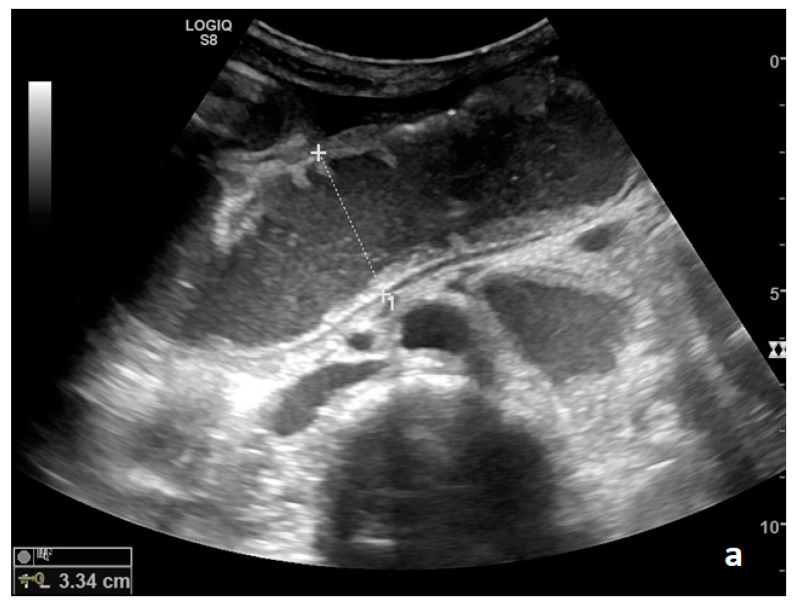

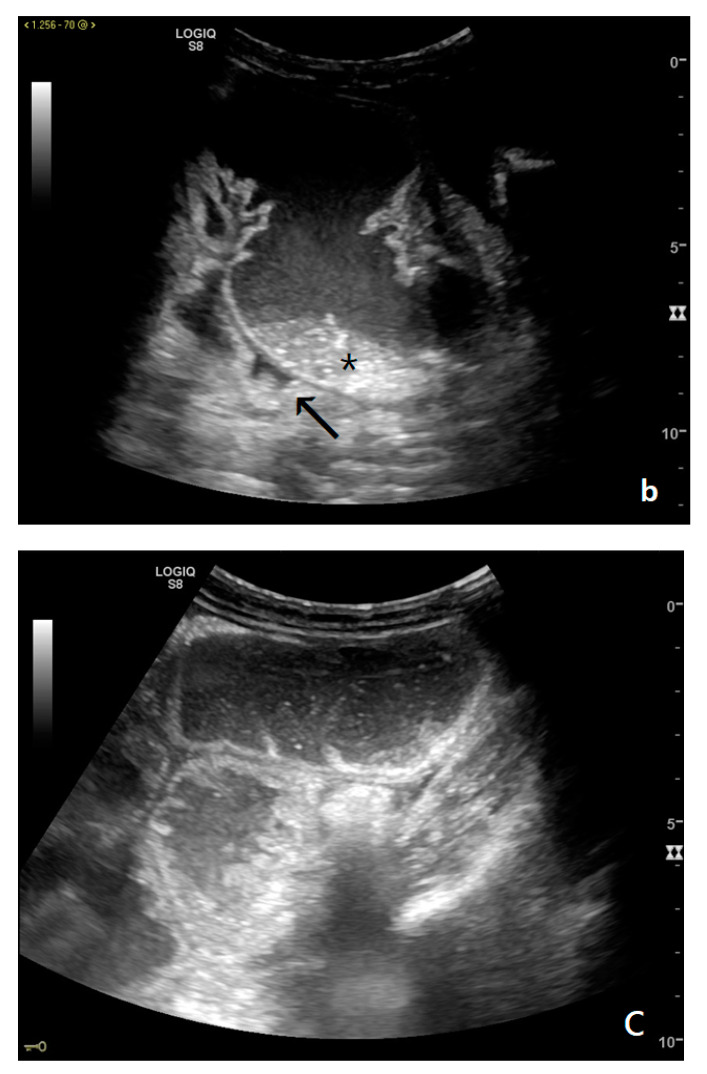

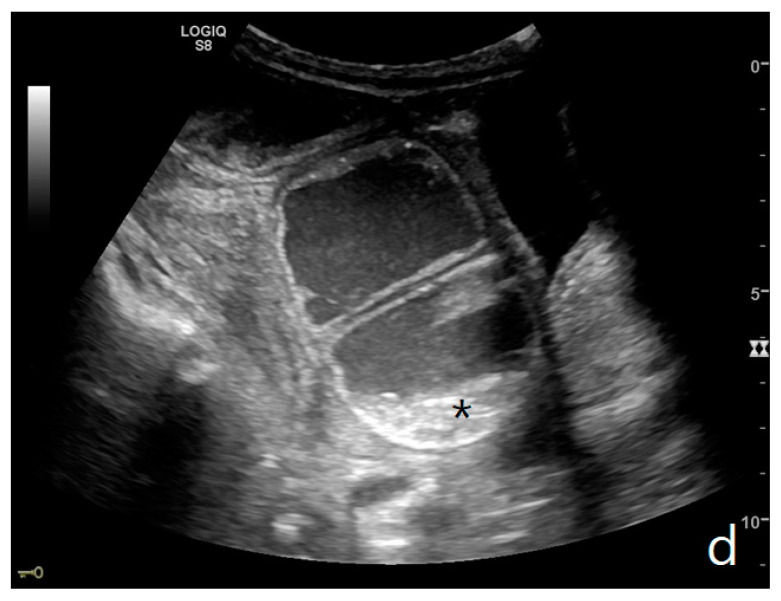
A complicated SBO in a 69-year-old male with gastric cancer and peritoneal carcinosis. Ultrasound images show long (**a**) and axial (**b**) evaluations of a fluid-filled, dilated small bowel loop with hyperechogenic floating material (shown with an asterisk) (**b**,**d**). Bowel peristalsis was absent. Mild parietal and valvulae conniventes thickening are present (**c**,**d**). Downstream loops present normal caliber (bowel jump diameter). Free fluid is interposed between bowel loops (black arrow) (**b**).

**Figure 5:**
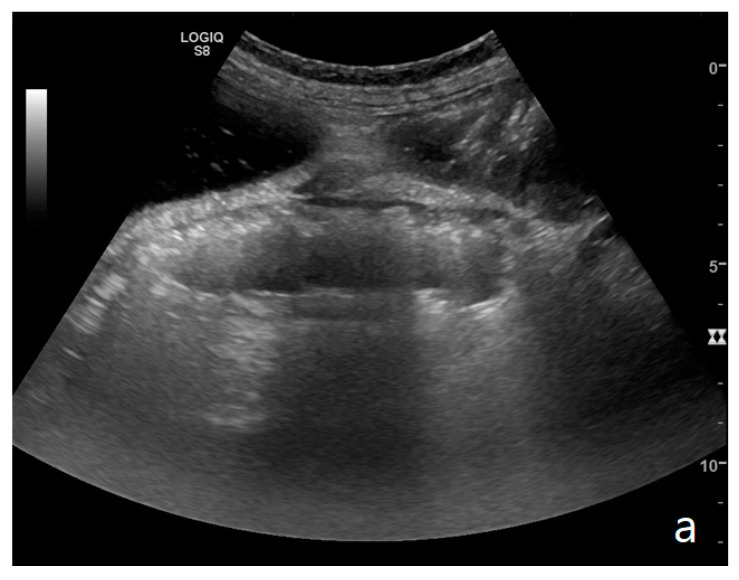

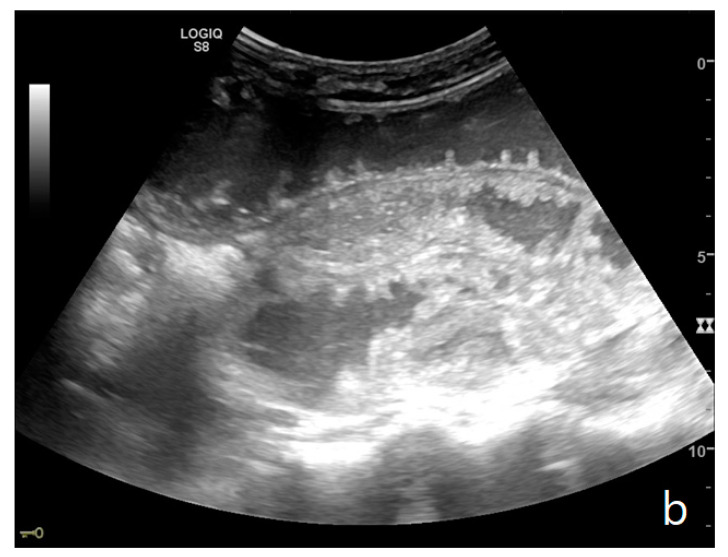
A complicated SBO presenting fluid-filled bowel loops with thickened walls with a stratified echo pattern (**a**) and thickened valvulae conniventes (**b**). Free fluid in the abdominal cavity was detected (**b**). Tail comet artifacts for air-fluid levels are visible (**a**). At the time of surgery, the bowel loop was necrotic.

Parietal changes are characterized by the presence or absence of parietal and valvulae conniventes thickening and parietal wall stratification. The evaluation of parietal changes follows a dichotomic diagnostic process based on the reference values (normal thickness 1–3 mm, wall thickening >3 mm, thinned walls <1 mm): thickened walls/valvulae conniventes (YES/NO) or thinned walls (YES/NO) (Figure 3a,b) [29,30,35]. Although ultrasound allows us to identify the five concentric layers of the intestinal loops, this evaluation is not applied in the diagnosis of SBO. In practice, the evaluation is limited to the presence or absence of parietal stratification (two-layer double halo sign or three-layer target sign) (Figure 4a–d and Figure 5a,b) [29,35,42].

The valvulae conniventes (Kerckring valves, circular folds) are permanent folds composed of mucosa and submucosa that project into the intestinal lumen and are clearly visible in the case of fluid distension (keyboard sign) [31]. At an early stage of SBO, it is not uncommon to see the valvulae in the upstream loop appearing more numerous and closer to each other. As the occlusive state continues, the loop upstream of the obstructive fulcrum becomes weaker, bowel walls appear thin, and the folds flatten (Figure 2a and Figure 3a,b). The upstream loops more distant from the obstructive fulcrum may still present peristalsis, albeit reduced and ineffective. In complicated ileus, with the onset of vascular loop distress, the walls and valvulae become thicker and weaker due to parietal edema and venous stasis, with possible dramatic parietal necrosis and subsequent perforation (Figure 4a–d and Figure 5a,b) [12,43].

#### 3.2.3. Ancillary Signs

Ancillary signs are useful in increasing diagnostic confidence.

These refer to the ‘bowel loop jump’—the visualization of two groups of loops with a clear difference in size (‘caliber jump’) or peristalsis (‘kinesis jump’). In both cases, a comparison should be made between the loop upstream and that downstream of the obstruction [12].

## 4. Conclusions

Ultrasound is a highly accurate imaging method for the diagnosis and staging of mechanical ileus of the small bowel, and the ultrasound findings fully reflect the evolutionary patterns of the disease. Moreover, ultrasound can be used to evaluate intestinal peristalsis in real-time, playing a key role in diagnosis and patient monitoring.

In addition, a patient with an ultrasound diagnosis of mechanical ileus occlusion can and should be admitted to the surgical ward.

Currently, contrast-enhanced ultrasound (CEUS) for the assessment of loop vascular distress is still considered level II imaging and is not widely used since it is not always able to detect the loop immediately upstream of the obstructive fulcrum, which is the subject of ultrasonographic study [44,45]. Ultrasonography is also widely used in the monitoring and follow-up of patients undergoing conservative treatment, allowing the assessment of loop distension and the resumption of peristalsis [11]. Even if it cannot be considered a substitute for level II imaging, such as CT, since it is not panoramic and not always able to identify the cause and loop vascular damage in all patients, it still plays a validated role as the first screening examination for confirming or excluding mechanical ileus, allowing a rapid bedside diagnosis and immediate inclusion of the patient in a diagnostic and therapeutic process [11,31,37,38,46].

## Figures and Tables

**Table 1:** Ultrasound criteria for small bowel obstruction (SBO) diagnosis.

	Simple	Decompensated	Complicated
**Bowel Loop Diameter**	Increased	Increased	Increased
**Parietal Thickness**	Normal	Normal or Increased	Increased
**Valvulae Conniventes**	Not Thickened	Not Thickened	Thickened
**Peristalsis**	Present and/or Hyperkinetic	Decreased	Absent
**Free Fluid**	Absent	Present	Present

**Table 2:** Ultrasound signs of SBO.

**Diagnostic Criteria**	
Loop Dilation	>2.5 cm
Kinesis Alterations	Altered Hyperkinesis (Early SBO) Hypokinesis Akinesis
**Staging Criteria**	
Free Fluid	
Parietal Alterations	Parietal and Valvulae Conniventes Thickening Parietal Wall Stratification
Ancillary Signs (Increased Diagnostic Confidence)	‘Caliber Jump’ ‘Kinesis Jump’

## Data Availability

All analyses were based on previously published studies. No new data were created in this study. Data sharing not applicable.
